# From the Wet Lab to the Web Lab: A Paradigm Shift in Brain Imaging Research

**DOI:** 10.3389/fninf.2019.00003

**Published:** 2019-03-01

**Authors:** Anisha Keshavan, Jean-Baptiste Poline

**Affiliations:** ^1^Department of Speech and Hearing, Institute for Neuroengineering, eScience Institute, University of Washington, Seattle, WA, United States; ^2^Faculty of Medicine, McConnell Brain Imaging Centre, Ludmer Centre for Neuroinformatics and Mental Health, Montreal Neurological Institute and Hospital, McGill University, Montreal, QC, Canada; ^3^Henry H. Wheeler Jr. Brain Imaging Center, Helen Wills Neuroscience Institute, University of California, Berkeley, Berkeley, CA, United States

**Keywords:** neuroimaging, open science, infrastructure, web browser, collaboration, communication

## Abstract

Web technology has transformed our lives, and has led to a paradigm shift in the computational sciences. As the neuroimaging informatics research community amasses large datasets to answer complex neuroscience questions, we find that the web is the best medium to facilitate novel insights by way of improved collaboration and communication. Here, we review the landscape of web technologies used in neuroimaging research, and discuss future applications, areas for improvement, and the limitations of using web technology in research. Fully incorporating web technology in our research lifecycle requires not only technical skill, but a widespread culture change; a shift from the small, focused “wet lab” to a multidisciplinary and largely collaborative “web lab.”

## 1. Introduction

The internet is ubiquitous and infiltrating every aspect of our lives by way of the web browser. Desktops, tablets, and cell phones have web browsers, but also televisions, game consoles, wristwatches, cars, glasses, and even refrigerators can effortlessly display all the information that resides on the internet. Information that, in theory, includes nearly all scientific knowledge.

The web browser has transformed our scientific practices, by giving us access to an almost infinite information resource. It provides a flexible and immediate platform for publishing research products. It gives us access to powerful computing platforms and databases. It enables us to collect large amounts of data from many people (e.g., citizen science). It is absolutely essential for communication and scientific collaboration. And above all, its main strength is its transportability; science, particularly in computational fields such as neuroimaging, can be performed anywhere (given a speedy internet connection).

Scientific collaboration is becoming increasingly important as computing technology enables us to rapidly collect and analyze data. The result of this data deluge is that we have an increased need for interdisciplinary, collaborative research. A combination of scientists with domain specific knowledge and those with a intimate grasp of computer science, data wrangling, and statistics/machine learning are needed to fully capitalize on the potential of large datasets.

We have witnessed enormous leaps of scientific knowledge that were a direct result of large scale collaborations, like the Human Genome Project, the Large Hadron Collider, ITER (research in nuclear fusion), and LIGO (to measure gravitational waves) to name a few. And it was primarily because of a large scientific collaboration at CERN where one of the most transformative technologies of the late 21st century was born: the World Wide Web.

Around the same time as the invention of the web came the invention of functional magnetic resonance imaging (Ogawa et al., 1990) in 1990, which revolutionized neuroscience research on brain-behavior relationships. Enabled with the ability to image brain function, neuroimaging researchers have been collecting vast amounts of data to answer more complex questions about the relationship between brain structure and function. And as a result, neuroimaging researchers are collecting large amounts of data, and encountering the same roadblocks and bottlenecks that come with any “big data” science. Here, we propose that by more deeply incorporating web technology into the lifecycle of neuroimaging research, we can not only accelerate neuroscience discoveries but also develop and test novel neuroscience questions. In the following sections, we discuss the paradigm shift that web technology brings to the scientific research lifecycle in terms of two main principles: collaboration and communication.

In addition, the use of web technology should have an impact on today's reproducibility crisis (Collins and Tabak, 2014). It has become clear in several fields of the life sciences that our current research practices are not best adapted to the production of robust and replicable results. Web technologies with their capacity to scale are key for the emergence of solutions to this crisis.

## 2. Collaboration

### 2.1. Data Sharing and the Web

One may remember the first attempts at data sharing in functional neuroimaging, the fMRI data center (Van Horn and Gazzaniga, 2013), and the difficulty of getting and reusing data sent over on compact discs or DVDs. Creating a culture of data sharing has many advantages: it can lead to more rapid scientific discovery for basic science and clinical research, can improve data quality, reduce costs, and improve reproducibility, and is in some cases a requirement made by funding agencies (Poline et al., 2012; Poldrack and Gorgolewski, 2014; Madan, 2017a). Some researchers argue that it is an ethical imperative (Brakewood and Poldrack, 2013) to maximize a subject's contributions, especially in clinical trials (Bauchner et al., 2016). But just because the data is shared, it doesn't mean the data can be found.

First and -possibly- foremost, browsers are the doors to the four principles of FAIR (Findable, Accessible, Interoperable, and Reusable), a set of guidelines developed by stakeholders in academia, industry, and funding agencies to promote data reuse (Wilkinson et al., 2016). We review them briefly here in the context of the web technology:

1. Findable: In order for scientists to discover data that may be of use for their research questions, datasets need to be indexed within a central database server, with appropriate metadata such that search engine algorithms can efficiently perform queries, and most importantly, with a browser-based user interface for researchers to submit queries and display results.
2. Accessible: the standard HTTP protocol used by browsers and web servers is open, free, and can provide authentication if needed.
3. Interoperable: all browsers speak the same language, regardless of their base operating system. Data description should adopt standards and convention to enable reuse across datasets, for instance through linked data technologies (Berners-Lee, 2009).
4. Reusability requires critically a community effort, to define relevant metadata and to standardize metadata reporting. This can be streamlined with web interfaces.

A key feature of the FAIR principles is that when possible they should be applicable not only to humans, but also to machines. For instance, datasets should be findable by “bots” by being tagged with the appropriate machine-readable metadata.

Neuroimaging groups have developed web portals that make it easy for other researchers to query, explore, contribute, and share both raw data and derived data. The COINS web platform (Scott et al., 2011) provides data management tools, an intuitive user interface, and was built with an emphasis for PHI security and multisite collaborations. The LORIS platform (Das et al., 2012) includes a web portal for data management and data quality control with neuroimaging viewers. The LONI Image Data Archive (LONI-IDA) is a long-term, centralized, HIPAA-compliant relational database archive for researchers to upload and share their data (Van Horn and Toga, 2009); as of this writing, the LONI-IDA has provided over 50 million downloads and over 1 million uploads to the archive. Web application such as these reduce the technical overhead to find, share, and aggregate data, and should ideally become standard practice for all large data collection efforts in neuroimaging.

The accessibility (FAIR-ness) of derived data is key to meta- and mega- analyses. A prominent example is the ENIGMA project (Thompson et al., 2014), which disseminated standardized analysis scripts to be able to co-analyze (e.g., a mega-analysis) a set of individual center's results, by sharing derived data rather than raw data. A mega-analysis strategy is especially optimal in cases where raw data sharing is not feasible. For task and resting state fMRI, the NeuroVault (Gorgolewski et al., 2015) web application enables scientists to upload fMRI statistical maps (e.g., derived data) in the standardized MNI space, and link to their publications; this platform includes both volume and surface-based visualization, and can enable more accurate meta- and mega-analyses. For diffusion imaging, the Automated Fiber Quantification (AFQ) package (Yeatman et al., 2012) has an associated web-viewer (Yeatman et al., 2018) and vault^1^ to easily share derived AFQ data in a standardized format. Building software that returns derived data in standardized formats and lowers barriers to sharing these derivatives with the neuroimaging community will facilitate meta- and mega- analyses in future years.

In the past, sharing data was a technical challenge (Van Horn and Gazzaniga, 2013); now, it is easier to share data even if the data are not part of a large consortium. The OpenNeuro web application enables researchers to upload and share their neuroimaging data as long as the data follow a community-developed standard to organize and describe neuroimaging datasets called the Brain Imaging Data Structure (BIDS) (Gorgolewski et al., 2016). Adopting standards for how data are stored enables sharing by reducing the overhead needed to curate heterogeneous datasets, and therefore promotes interoperability and reusability of data (Tenopir et al., 2011). Examples of standardized data formats outside of the neuroimaging field include the Open Geospatial Consortium (Castronova et al., 2013) and the Ecological Metadata Language (Fegraus et al., 2005).

In general, the FAIR principles do not stipulate how data sharing should be incentivized. The adoption of FAIR principles requires financial support as well as community adoption. While the OpenNeuro project has been funded by the NIH^2^, the BIDS standard that it relies upon is, importantly, starting to be adopted by a wide community. The standard has recently been endorsed by the International Neuroinformatics Coordinating Facility (INCF)^3^, and is recommended by several journals. Funding agencies (e.g., the Wellcome Trust^4^) are increasingly asking that a wider set of research products are shared with the community to increase reuse and maximize the funding impact on research. The set of tools that facilitate the conversion of small datasets to BIDS format is also growing (see the BIDS starter kit^5^), which may mitigate the need for long-term funding. Concurrently, training material to educate researchers to adopt the BIDS format is being actively developed by ReproNim (e.g the “FAIR data” module^6^).

In the genomic community, the Bermuda principles (Contreras, 2011) led to the establishment of few large public databases, but the brain imaging community has been less unified. This led to a variety of large or small initiatives, such as ADNI (Mueller et al., 2005), BIRN (Keator et al., 2008), BrainMap (Laird et al., 2011), INDI (Mennes et al., 2013), OpenfMRI (Poldrack et al., 2013), OMEGA (Niso et al., 2016), OpenNeuro (Gorgolewski et al., 2017a), Schizconnect portal(Wang et al., 2016), Healthy Brain Network (Alexander et al., 2017) to name a few [for more, see (Eickhoff et al., 2016)], and more recently the funder-based National Data Archive. Specialized tools to discover these resources and their content are improving fast [see for instance Scicrunch (Grethe et al., 2014)].

Efforts have begun in the neuroimaging community to create centralized resources to find openly released neuroimaging datasets. A very simple yet valuable collection was collaboratively compiled on the social coding platform Github^7^. OpenMorph,^8^ (Madan et al., 2018), is a curated list of open access datasets that can be used to study brain morphology. It includes sample sizes, types of MRI modalities, the associated publications and a link to each project's web portal to download the data. Anyone can contribute to this collection by creating a GitHub^9^ account and editing the document. The DataLad (Halchenko et al., 2018) project has developed a crawler to index the data from various scientific data portals for a unified interface from which to download these datasets from the command line interface on their computers. DataLad also hosts a web application to interactively explore the various datasets that have been indexed. We hope to see more aggregation of open neuroimaging datasets in the future, with accessible web interfaces to query and explore all our resources.

**Figure 1:**
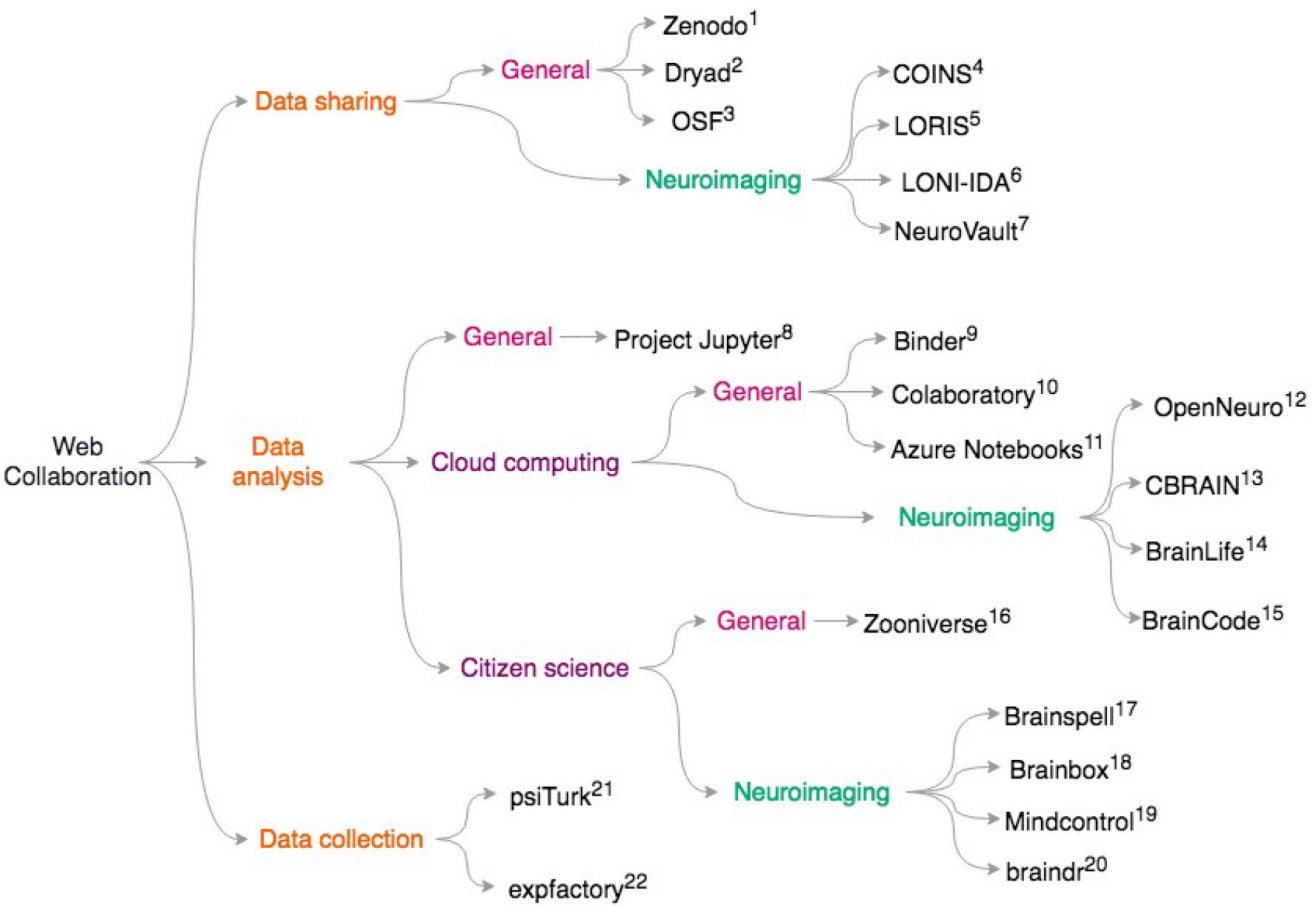
Overview of discussed collaborative scientific web tools. General resources for data sharing include. (1) Zenodo https://zenodo.org; (2) Dryad: https://datadryad.org/; (3) OSF: https://osf.io; Neuroimaging specific data sharing resources include (4) COINS (Scott et al., 2011) (5) LORIS (Das et al., 2012) (6) LONI-IDA (Van Horn and Toga, 2009) (7) NeuroVault (Gorgolewski et al., 2015) For general data analysis: (8) Project Jupyter (Ragan-Kelley et al., 2014; Kluyver et al., 2016) To access cloud resources with Jupyter notebooks, try: (9) Binder https://mybinder.org/; (10) Colaboratory https://colab.research.google.com/notebook; (11) Azure Notebooks https://notebooks.azure.com; For neuroimaging specific cloud computing, see (12) OpenNeuro (Gorgolewski et al., 2017a), https://openneuro.org; (13) CBRAIN (Sherif et al., 2014) (14) BrainLife (Hayashi and Pestilli, 2017), https://brainlife.io; (15) BrainCode (Vaccarino et al., 2018), https://www.braincode.ca/; For data analysis with citizen science, see (16) Zooniverse (Simpson et al., 2014), https://zooniverse.org; and for neuroimaging-specific projects, see: (17) Brainspell (Badhwar et al., 2016), https://brainspell.org; (18) BrainBox (Heuer et al., 2016), http://brainbox.pasteur.fr; (19) Mindcontrol (Keshavan et al., 2017a), https://mindcontrol-hbn.herokuapp.com; (20) braindr (Keshavan et al., 2018), https://braindr.us; For behavioral experiments, web services such as (21) psiTurk (Gureckis et al., 2016), https://psiturk.org; (22) expfactory integrate with Amazon mTurk. (Sochat et al., 2016), https://expfactory.org.

More generally, platforms like Zenodo (https://zenodo.org), Dryad https://datadryad.org/, and the Open Science Framework https://osf.io give researchers generous storage for their datasets and assign digital object identifiers (DOIs) to datasets. This means that researchers who primarily collect data can get credit via citations, potentially alleviating concerns about “research parasites” (Longo and Drazen, 2016) that prevent some from openly sharing data. Our scientific culture is in part a roadblock to data sharing (Tenopir et al., 2011). Ideally, moving away from placing importance on only the first and last authors during grant and career reviews may incentivize data sharing and large collaborations. It is clear that technical challenges are not the only barrier to data sharing; we discuss the social and ethical challenges with data sharing in the “pitfalls” section. For an overview of the resources on data sharing, data analysis, and data collection, see Figure 1.

### 2.2. Collaborative Work and the Web

#### 2.2.1. Collaborative Data Analysis Through the Web

Data, albeit the foundation of most work, is only the first element of a research project. The reusability of other research products such as software, libraries, scripts, and pipelines or workflows, has traditionally been poor, with the exception of a few neuroimaging software packages [e.g., SPM (Friston et al., 1994), FSL (Smith et al., 2004), and Freesurfer (Fischl, 2012)]. With a greater ease of dissemination and search of these objects, research is entering a phase of accelerated efficiency, providing building blocks for fast construction of a new analysis. Todays researcher in neuroimaging is able to search for and download an entire software environment in a Docker^10^ container and launch complex pre-processings and analyses. Neurodocker (Kaczmarzyk et al., 2018) makes it possible in a single command line to create an environment with all necessary software specific version for an analysis. Reprozip (Chirigati et al., 2016) makes it possible to trace all the dependencies of a single command and create reusable packages that rerun the exact command, even on a different system. fMRIprep (Esteban et al., 2019) and MRIQC (Esteban et al., 2017) provide environments for fMRI preprocessing or MRI quality control. Work that may have taken a post-doc or a graduate student a few months can take now a few days if not a few hours. This order of magnitude acceleration factor has been made possible because (1) these projects are often highly collaborative and often will have inputs from tens of individuals leveraging social coding platforms (e.g., Github), and (2) the communication of the technologies and repositories through web based platforms.

Cloud computing provides unlimited, scalable, computing resources (provided enough financial resources), but can be difficult to interface with because it requires specialized expertise. Through web interfaces, cloud computing can be made accessible such that domain specific researchers can reap its full benefits. OpenNeuro (Gorgolewski et al., 2017a), currently hosted on Amazon Web Services, enables researchers to upload BIDS-compatible datasets and then run analyses via BIDS-Apps (Gorgolewski et al., 2017b) on the AWS cloud for free, given that the data is publicly shared after a certain grace period. The Canadian Brain Imaging Research Platform (CBRAIN) web platform (Sherif et al., 2014) can bring together heterogeneous data sources and compute grids into one, secure web interface. The BrainLife^11^ (Hayashi and Pestilli, 2017) web application is in development to provide researchers with an intuitive interface to cloud computing resources, enable data sharing, and the publishing of results with clear provenance. The Brain-Code (Vaccarino et al., 2018)^12^ web portal and data management/analysis platform aims to foster collaboration and data discovery across various clinical brain disorders.

The Jupyter project (Ragan-Kelley et al., 2014; Kluyver et al., 2016) has been actively developing a web-based scientific notebook interface for various programming languages (Julia, Python, R, and more). Researchers can interact with various programming kernels on a web interface that can be deployed locally, or on the cloud. The resulting notebook can be shared as a website, with not only code displayed but also the resulting figures, and associated documentation that is formatted in Markdown, which can also render equations. The Jupyter notebook comes with the ability to write interactive widgets, such as javascript-based sliders that let users explore various parameter spaces of the functions they write. Interactive plotting libraries, like Plotly^13^ can be integrated within the Jupyter notebook, enabling researchers to create rich, interactive data visualizations. The Binder^14^ project, as of this writing, provides a free service to host instances of Jupyter notebooks on the cloud. Azure notebooks^15^ and Google Colaboratory^16^ also provide similar notebook hosting services. Currently, Colaboratory provides access to GPUs instances, which are incredibly useful for deep learning projects. Services that enable easy deployment of notebooks and their associated computing environments will vastly improve the transportability of research objects; we therefore encourage neuroinformatics researchers to take advantage of these web services.

#### 2.2.2. Collaborative Writing on the Web

In the past, collaboratively preparing manuscripts might only have been possible with those in a scientist's immediate vicinity. With the web browser, email drastically improved the collaborative writing process, but it is still a slow, serial process of emailing documents back and forth. Google Docs^17^ was a breakthrough web application that parallelized the manuscript preparation process by enabling multiple authors to simultaneously write, edit, comment, and even chat with each other. Version control, tracking changes, and generous free cloud storage means researchers are much less likely to lose their work. Microsoft Word, the most widely used software for preparing manuscripts, offers an “edit in the browser” feature for realtime collaborative editing^18^. For reference management, Paperpile^19^ interfaces nicely with Google Docs. For those who prefer to prepare manuscripts with LaTeX, services such as Overleaf^20^ and Authorea^21^ compile latex on the cloud, removing the technical overhead of setting up latex locally and compiling the document. Collaborators who are less familiar with LaTeX can now easily contribute to these manuscripts. See Table 1 for a summary of collaborative writing web applications.

**Table 1:** Summary of collaborative tools for writing manuscripts and code on the web.

**Name**	**URL**	**Comment**
Google Drive	https://drive.google.com	Write manuscripts, spreadsheets, etc
Office 365	https://office.com	Write manuscripts MS Word online.
Paperpile	https://paperpile.com	Reference manager for google docs
Overleaf	https://overleaf.com	Write manuscripts (LaTEX)
Authorea	https://authorea.com	Write manuscripts (LaTEX, HTML)
GitHub	https://github.com	Write code
GitLab	https://gitlab.com	Write code
Travis-CI	https://travis-ci.com	Test code (links to GitHub/Lab)
Circle-CI	https://circleci.com	Test code (links to GitHub/Lab)

GitHub^22^, “the social coding platform”, has simplified and improved the collaborative writing of software. Github provides a visual representation of the somewhat complicated git version control system. GitHub repositories contain the full codebase for a project, all the changes that have been made, and who made them (via git). Users can“Fork” GitHub repositories, which makes a copy of the code to their account. They can then make changes to the code and send the changes back to the original repository via “Pull Requests,” which begins a discussion thread for others to comment on the code (called a code review). GitHub also provides an “Issues” page for each repository, where users can discuss any issues and ask the community for help. Continuous integration software testing can be automatically run on the cloud once changes to the code are pushed to GitHub, by web-hooks to services like Travis CI^23^ and Circle CI^24^, which provide a generous free tier for open source projects. GitHub repositories can also host static websites; this is extremely useful for hosting code documentation. GitLab^25^ is an open source alternative to GitHub, which can be deployed by researchers in cases where they need a private git web application. Many open source neuroimaging tools are built collaboratively on GitHub, such as Nipype^26^ (Gorgolewski et al., 2011), Dipy^27^ (Garyfallidis et al., 2014), and Nilearn^28^ (Abraham et al., 2014), to name a few. By developing open source neuroimaging software packages on social coding web interfaces, researchers are able to engage a much larger community of contributors than would have been possible in the earlier days of the web.

#### 2.2.3. The Web for Mass Collaboration: Citizen Science and Crowdsourcing

The web browser is particularly well suited for citizen science and crowdsourcing; this is becoming necessary as neuroimaging datasets grow, and data analysis bottlenecks arise when massive amounts of data need visual inspection. In the astronomy community, the Galaxy Zoo (Lintott et al., 2008) web application was successful at engaging citizen scientists in visually classifying galaxies. This project evolved into a more general citizen science platform called the Zooniverse (Simpson et al., 2014), which enables researchers from any domain to engage citizen scientists in annotating their data. In the neuroscience field, EyeWire (Kim et al., 2014) and Mozak (Roskams and Popović, 2016) have gamified the tracing of neurons. The EyeWire project was able to engaged over 100,000 citizen scientists from all over the world to collaboratively trace the neurons of the human retina. Such a massive engagement of collaborators would not have been possible without the web browser.

The neuroimaging community is just beginning to engage citizen scientists as a resource in our data analyses. The Brainspell (Badhwar et al., 2016) web application was developed to manually annotate fMRI coordinate tables that were automatically extracted by Neurosynth https://neurosynth.org (Yarkoni et al., 2011), which itself is a web application to perform coordinate-based fMRI meta-analyses. BrainBox (Heuer et al., 2016) and Mindcontrol (Keshavan et al., 2017a) are web applications to annotate MRI volumes (e.g., to edit segmentations). Recently, a mobile-optimized and gamified web application called braindr (Keshavan et al., 2018) was developed to perform quality control on images from the Healthy Brain Network initiative. At the time of this writing, braindr has engaged over 400 citizen scientists and over 100,000 annotations. Image labels were aggregated by weighting citizen scientists based on how well their ratings matched an expertly labeled “gold standard” subset of images. A deep learning network was then trained from these aggregated labels to automatically rate image quality to near perfect accuracy. Hybrid human-computer approaches for quality control seem the most promising (Esteban et al., 2018), such as “triaging” image reviews based on machine-learning output probability scores for Freesurfer image segmentation as in Klapwijk et al. (2018). Whether citizen science applications can go beyond quality control and toward more complex tasks like image segmentation and registration remains to be explored.

The cognitive science and psychology communities often utilize paid crowdsourcing web platforms like Amazon Mechanical Turk (mTurk) to run behavioral experiments with large, diverse populations. The psiTurk (Gureckis et al., 2016) and ExpFactory (Sochat et al., 2016) frameworks enable scientists to interface with mTurk and create reusable web-based psychology experiments. For image processing, the quanti.us (Hughes et al., 2018) platform can be used to interface with mTurk to crowdsource the segmentation of biological images. In neuroimaging, Ganz et al. (2017) showed it was feasible to crowdsource the detection of Freesurfer (Fischl, 2012) cortical surface delineation errors on mTurk. We expect to see more utilization of citizen science, gamification, and paid crowdsourcing platforms in neuroimaging research, and there are still many open questions about which strategies (citizen science vs. paid crowdsourcing) and task designs are better suited for various analyses, as well as how to properly acknowledge the contributions of citizen scientists [see (Hunter and Hsu, 2015) for a proposed method].

### 2.3. Pitfalls

Even though the benefits of the web browser for scientific collaborations are evident, using the web for our research comes with some drawbacks or difficulties. Collaboration requires the sharing of data, and while some argue that data sharing is an ethical imperative (Brakewood and Poldrack, 2013; Bauchner et al., 2016), one must consider the risks of reidentification of our subjects, particularly for clinical research. True deidentification is difficult because of linked metadata (Narayanan and Shmatikov, 2008; de Montjoye et al., 2015). For example, in Narayanan and Shmatikov (2008), researchers identified pseudo-anonymized Netflix users by linking data with metadata from another website (IMDB). In de Montjoye et al. (2015), researchers proved that pseudo-anonymized credit card data could be reidentified provided just four spatiotemporal points. Research in differential privacy (Sarwate et al., 2014) might alleviate some of these risks; regardless, it is important that subjects are made aware of the risk in the consent process. The Open Brain Consent website^29^ is a collaborative effort to provide resources that aid researchers in the IRB process for sharing data, writing the consent form, and tools for the anonymization of neuroimaging data.

Legal obligations concerning personal data handling are evolving and the recent European Union General Data Protection Regulation (GDPR) will likely change the requirements for participants control over their personal data. This will need to be considered at all stages of the research data lifecycle. While a full discussion on the legal and ethical aspects of data dissemination and reuse is out of the scope of this article [see for example (Marelli and Testa, 2018) on the GDPR] it is clear that legal and regulatory constraints are going to shape the implementation and use of web based data dissemination and retrieval tools, and this will require increased attention and human resources in the future. The challenge will be to constantly adapt our infrastructures and practices to the new regulations, which will require continuous software development.

Another drawback of using web technology for collaboration, in terms of sharing data, accessing cloud resources for analysis, and distributing work, is bit rot (Baker et al., 2006; Cerf, 2011). Bit rot refers to the eventual degradation of information stored on electronic media; for example, information stored on floppy disks is likely not accessible for most of us. Web technology is advancing rapidly: the browsers we use now look nothing like they did a decade ago. Some websites that were built in the past do not work with modern browser technology, and most websites from the past are not available to us anymore. A decade from now, many of the links presented in this article may no longer exist. Servers cost money, and domain names are charged annually. Software needs to be consistently maintained to be compatible with current technology. Efforts such as the Internet Archive^30^ and Digital Object Identifier (DOI) system are working to preserve the information on the web, and in the case of DOI, provide persistent links to our research articles. But we need to work with funding agencies to ensure we have the resources to maintain scientific output, outside of our research articles, that depend on web technology. We also need to work with publishers to ensure our full scientific output, including the web technology that is used to produce it, can be fully preserved.

Finally, web-based research depends on a stable and fast internet connection. Such infrastructure may not be available to scientists in developing countries, which further drives inequalities and will decrease the diversity of our scientific community. It is important to keep this in mind when designing web applications, by optimizing websites for slow internet connections, and building offline support.

## 3. Communication

Scientific work in the public imagination is still often thought to be a rather solitary activity of independent individuals, sometimes attracting introverted personalities. But actual scientific work is largely communication, where a large proportion of time is spent thinking of the best way to communicate research to collaborators, to scientific communities, to the public, and to funders. Different scientific fields have different levels of interdependencies. A researcher in a specialized mathematical subfield like non Riemannian geometry could be mostly working on their own, but fields like neuroscience or the biomedical sciences are highly multidisciplinary. The ability to absorb and reuse research from other laboratories is most often critical for progress, as the systems studied are both too complex and too interdependent to be understood by individuals or single labs. While conferences and in-person meetings are traditional methods for communicating research, the web now expands scientific communication to a completely new level, by removing time delays and scalability constraints. Now, even social network communication tools are used for the benefit of scientific communication.

### 3.1. Local Networks Communication

The small or medium size laboratory structure [5–15 people (Conti and Liu, 2015; Cook et al., 2015)] is still the predominant basic research structure in universities and research institutes, and these are mostly set up such that in person meetings are practical. Nevertheless, it is common that one or several members of the laboratory are temporarily located in another institution or building and the meeting will occur through web video conferences. The number of companies proposing free or paid services that may include capacity to share documents has multiplied during the past few years (the authors count at least 7 web video conference systems as of today, for instance Zoom^31^, Webex^32^, BlueJeans^33^, Skype^34^, Google Hangouts^35^, appear.in^36^, GoToMeeting^37^, etc, as well as project management systems such as Trello^38^ or Asana^39^), allowing for unprecedented efficiency even in local communication. A key aspect of some of these communication tools is their capacity to record the meeting (audio-video) permitting delayed communication and traceability of discussion points, ideas or decisions, as well as scaling for larger groups. Another key aspect is that the use of these tools allow a group to immediately scale to non local members.

### 3.2. Scholarly Communication

A neuroimaging or neuroscience researcher's work is heavily influenced, if not directed by, the search for funding and progression in academia career. As these mostly still depend on the quality and number of publications, it is clear that publishing activity is central to a researcher's academic life.

The current publishing industry is still very much influenced by how this activity used to be at the turn of the twentieth century, at a time when manuscripts had to be manually typed and printed, and distribution of journals was achieved through mail. Today, the article remains a standard for scholarly communication, even though an increasing number of researchers realize that the actual scholarship may actually reside in the code and data rather than the article^40^. Jon Claerbout, a professor from Stanford University, argues that an article about a computational result is advertising, rather than scholarship. The actual scholarship is the full software environment, code and data, that produced the result (Donoho, 2010). The web has transformed the industry and is *de facto* the new media for scholarly communication, but somehow less rapidly and less radically than it could have. Most traditional journals are still shipping some printed copies of their editions, while a very large number of “on-line only” journals with an open access policy have emerged with a business model based on article processing charges (ACP), occasionally generating low quality content, but a highly profitable business (for a long list of questionable publishers, see the Beall's List^41^). We note that Beall's list does not necessarily have the level of granularity required as it can address general publishers rather than specific journals.

Even when the web is adopted as the communication media, the very large majority of the articles are based on HTML and PDF, with almost none of the modern visualization and interactive figure components that can be delivered by modern JavaScript libraries (e.g., D3.js^42^). In neuroimaging, a number of open source, browser-based visualization tools have been developed. Javascript brain viewers like BrainBrowser (Sherif et al., 2015), papaya.js^43^, XTK.js^44^ (Haehn et al., 2014), and AMI library (Bernal-Rusiel et al., 2017) enable researchers visualize neuroimaging data in the browser. Interactive, linked data dashboards have been built as outputs of neuroimaging software, like ROYGBIV^45^ (Keshavan et al., 2017b; Klein et al., 2017), AFQ-Browser^46^ (Yeatman et al., 2018), and MRIQC has a web-based viewer to visually inspect outputs (Esteban et al., 2017). The Open Anatomy Browser^47^ (Halle et al., 2017) hosts a variety of atlases with collaborative viewing. These tools have greatly simplified the process of building and sharing complex, interactive visualizations. For example, researchers may deploy an AFQ-Browser visualization of their data with two simple commands (afqbrowser-assemble, afqbrowser-publish). These interactive figures may go much beyond the convenience of a better view of the result; they allow to test for the potential robustness or sensitivity with data input or methods in a way that cannot be provided by static figures. In such a case, some parts of the scholarship need to be communicated by interactive figures, but few publishers are able to provide the infrastructure for hosting such “interactive articles”.

Recently, the rise of documents able to mix code and narrative such as R-markdown or Jupyter notebooks also provide researchers with new opportunities for communicating full fledged research objects. Some publishers already have embraced these new possibilities. For instance, eLife is working with Stencila^48^, designed to be documents that “…are self-contained, interactive and reusable, containing all the text, media, code and data needed to fully support the narrative of research discovery” to foster more reproducible and reusable research, see eLife. In the near future, systems such as Binder (Jupyter et al., 2018) will allow not only to publish and review the computational documents but also provide with a container and the environment for a fully re-executable publication. The new web tools are not only key to provide us with ways of publishing a more complete set of research objects, they also allow for new review workflows to be implemented. For instance, Frontiers developed a platform that intended to make the interaction between reviewers and authors more efficient. Tools such as https://web.hypothes.is/^49^ permit readers to annotate only specific parts of an article and may in the future be re-used by a review system. Such a review system could associate expert reviews and open community based reviews.

The web is also transforming how research communities meet for discussions by creating virtual conferences. A number of virtual conferences have been successfully organized in the past, removing the constraints of space and travels, while still allowing for questions and answer sessions monitored online (see for instance neuroscience-2018^50^). A recent twitter conference was recently organized (the Brain Twitter Conference) which could scale easily to tens of thousands of participants. These events are much easier to organize in a short time and less costly if not free for attendees. They also are possible to attend by all researchers independently of possible travel and funding restrictions and are only limited by time zone constraints. For example, Chris Madan advocates using Twitter for science in (Madan, 2017b), and see his associated blog post^51^ on this topic.

In the same spirit, global Brainhack events gather locally groups of neuroinformaticians who collaborate on software development projects, and are also sharing courses and seminars across locations. The latest Brainhack^52^ event took place in 16 countries and gathered more than 1000 participants in 5 different time zones. The University of Washington hosts various week-long summer schools or“hack weeks” (e.g., Astrohackweek, Geohackweek, and Neurohackweek) to promote education and training, tool development, community building, and interdisciplinary research by combining pedagogy with project-based learning (the“hacking”) around a specific domain (Huppenkothen et al., 2018). They found that this combination is particularly effective at fostering collaborations and promoting best practices. Through collaborative web applications like GitHub, the projects started at these hackweeks have continued even after the events ended, and have resulted in measurable scientific output [for details, see Huppenkothen et al. (2018)]. Data analysis challenges hosted by conferences or symposia like MICCAI^53^ bring researchers together to solve problems in the field, even if they cannot be present at the conference, and these groups collaboratively publish their results [for example see Commowick et al. (2018) for the results of a multiple sclerosis lesion segmentation challenge]. A curated list of biomedical image challenges can be found at https://grand-challenge.org/challenges/. We expect these types of events to be more frequent in the future, limiting the ecological, time, and cost impact of physical travels but offering the capacity for communication of research at a truly global scale.

### 3.3. Larger Public Communication

Ultimately, research needs to go beyond the scientists and will need to be disseminated to the larger public which, through taxes, is funding a large part of it (Illes et al., 2010). The field of neuroimaging necessitates costly acquisition devices (MRI, PET, E/MEG), and has been particularly well funded, not only because of its potential for neuroscience, but also because the ideas were communicated well to the public and to funders. Communication is now largely operated and achieved by social media platforms such as Twitter, LinkedIn, Facebook, and blog platforms, to name a few. To read more about the advantages and disadvantages of social media use for scientific communication, see (Bik and Goldstein, 2013). Online resources that teach how to effectively communicate science are provided by the Alda-Kavli Center for Communicating Science^54^. To consolidate the current consensus of knowledge, Wikipedia is probably the best resource; offering an introduction to functional magnetic resonance imaging through the consensus writing of many researchers (for example, see the Wikipedia article for fMRI).

**Figure 2:**
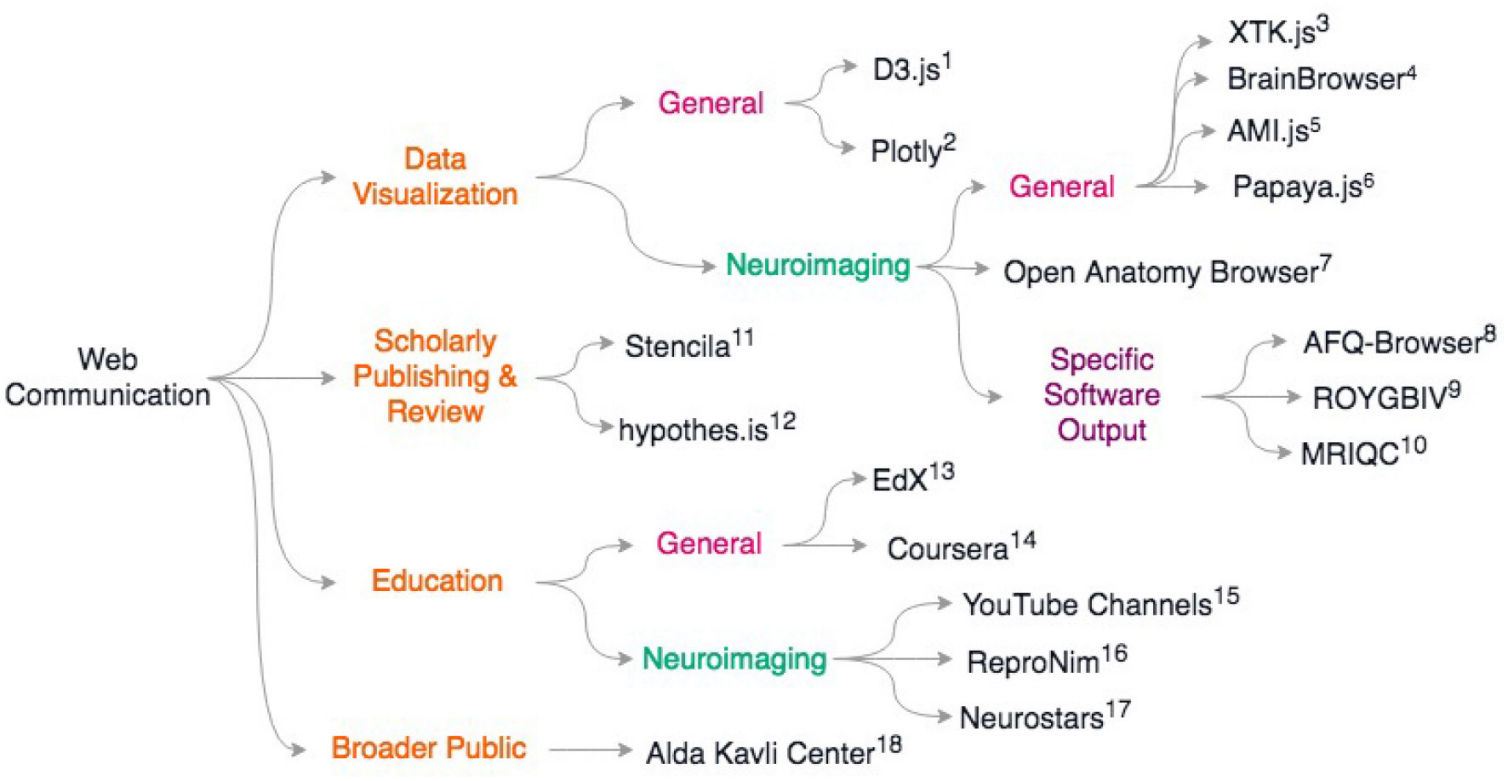
Overview of discussed scientific communication web resources. General resources for data sharing include (1) D3.js https://d3js.org; 2) Plotly: https://plotly.com/; General neuroimaging data visualization libraries include 3) XTK (Haehn et al., 2014) 4) BrainBrowser (Sherif et al., 2015) 5) AMI.js (Bernal-Rusiel et al., 2017) 6) papaya.js https://github.com/rii-mango/papaya; 7) Open Anatomy Browser (Halle et al., 2017) Some neuroimaging packages that release associated web-viewers: 8) AFQ-Browser (Yeatman et al., 2018) 9) ROYGBIV/Mindboggle (Keshavan et al., 2017b; Klein et al., 2017) 10) MRIQC (Esteban et al., 2017) For scholarly publishing and review: 11) Stencila https://stenci.la; 12) hypothes.is https://hypothes.is; In education: 13) EdX https://www.edx.org/; 14) Coursera https://www.coursera.org/; For neuroimaging-specific courses and resources: 15) YouTube channels of Dr. Jeanette Mumford and Dr. Dirk Ostwald 16) ReproNim training modules http://www.reproducibleimaging.org/; 17) Neurostars forum https://neurostars.org; Web resources for learning how to communicate to the general public: 18) Alda-Kavli Learning Center online resources https://www.aldacenter.org/AKLC

Last, but certainly not least, web based education platforms are also re-inventing how training is performed in neuroimaging. The standard in person courses are now often replaced by on-line material (see ReproNim^55^, Coursera online courses^56^, EdX online courses^57^), and a series of YouTube videos by Jeanette Mumford^58^) and also Dirk Ostwald^59^ as examples, amongst many good online materials. This allows laboratories to give some inverted classroom type of training by considering that formal lectures can be taken on-line but exercises or projects are best solved or supervised with direct interactions. It should be noted that on-line question and answer forums such as Neurostars^60^ (with a tagging system similar to stackoverflow^61^), NITRC, and software tool email lists, are also key for the training of young researchers and boost efficiency. For a review of scientific web communcation tools, see Figure 2.

### 3.4. Pitfalls

There are both limits and dangers associated with relying too much - possibly almost fully - on web technologies and browser enabled applications and workflows for research. Web communication does not necessarily allow the level of in depth interactions that are required to discuss a specific research question. In person meetings can be necessary both to organize projects and to advance the understanding of our scientific questions. In our experience, in person meetings are better at providing decision structures and at building trust, which are both necessary for the management of scientific projects.

Some of the dangers associated with the use of social media could also propagate to the scientific arena. For instance, while social media may be a great medium for quickly accessing or publicizing articles, it may also focus the attention on a specific cluster of the scientific community. This in turn may create research networks that are less permeable to different ideas, like a scientific echo chamber (Kim et al., 2017).

The immediate access to non -or poorly- peer-reviewed works may also amplify incorrect results that would not stand scrutiny under peer review. Take for example, a paper posted on the preprint server arXiv called “Automated Inference on Criminality using Face Images,” which received a lot of criticism from the scientific community^62^. Even though it was not peer reviewed, and as of this writing has not been published in a peer-reviewed journal, it nevertheless received a lot of alarmist press coverage. This can occur within the traditional literature, albeit at a slower pace. The neuroscience and public health communities are still contending with the spread of misinformation regarding a link between vaccines and autism (Del Vicario et al., 2016), despite the strong evidence to the contrary (Taylor et al., 2014). Scientific communication is our responsibility as scientists, not only to the scientific community but to the general public; we must be cautious of the immediacy of the web.

## 4. Conclusion

The way web technologies - and the browser as the window to these - are transforming scientific activity is still evolving. It is clear that an important part of research work will be on-line for the future PhD student, whether to acquire or disseminate knowledge, conduct an experiment, and collaborate with experts. This paradigm shift is already apparent with the advent of e-conferences and the use of social media in the neuroscience community. Some researchers now mostly rely on their Twitter feeds to learn about new and interesting studies, delivering more directed and rapid content than a traditional journal's table of contents. The browser brings the potential for massive online collaboration and more effective communication, but the web is still mostly an untapped resource in the neuroimaging and neuroscience fields.

Some scholars argue that we are having a reproducibility crisis. Many neuroimaging studies are found underpowered, and have reported possibly inflated effect sizes and unstable results (Yarkoni, 2009). We believe the browser can help, by connecting users to large, documented, and shared datasets through web portals, and by providing interfaces to upload, annotate, share and publish raw and derived data. This would result in a much broader pool of data that could be investigated and lead to more stable results, such as those from meta- or a more distributed, ENIGMA-style mega- analyses. These efforts should complete the FAIR principles, moving toward “Interoperable and Reusable” data, with community-defined documentation and metadata standards.

Replicating a study is complex because computing environments are difficult to transport to other systems. Works produced with tools that are not easily transportable to the web will be harder to communicate, and potentially less reproducible or re-usable by others. The analysis of a dataset performed on a local computer and producing figures as files on a local disk will need to consider all the hurdles of local storage, computational environments, and other technological challenges, to create robust software tools that work on all computational environments. Difficult installations limit the capacity to rapidly reuse the results. The web browser can help: the same analysis developed through a Jupyter notebook interface and running on the Binder service will be re-usable at no cost of transfer on either the producer and the receiver side. Considering the cost for an individual or lab to reproduce an analysis, collaborate on it, or re-use a component of it, should be a key question when working on a research project. In many cases, web technologies are the ideal solution.

We are experiencing a data deluge. As neuroimaging studies accumulate larger datasets, we encounter many new challenges in data analysis that we did not have with smaller datasets, both in terms of our capacity to consolidate datasets originating from various cohorts acquired on different scanners, and in terms of the sheer computational power needed to process very large datasets. Browsers, by interfacing with cloud computing infrastructures, can provide us access to an almost infinite resource of compute power. Data analyses that require visual inspection are unfeasible to scale; the browser provides the medium to collaborate with not only other experts, but also citizen scientists. Communicating insights from high-dimensional datasets is challenging, but the browser can host interactive data visualizations that can be easily shared. As a community we need to move toward developing browser-based tools to efficiently gain insights from large neuroimaging datasets.

The browser was built under egalitarian principles of free and open information exchange^63^, but scientific information is not completely free. Today, traditional scientific publishers are making unusually high profit margins and a large body of the literature is behind paywalls (Buranyi, 2017). This prevents text-mining and creates an unnecessary bottleneck to much needed meta-analyses (Van Noorden, 2012). In addition, research has become highly competitive [e.g., the famous adage, “publish or perish” (De Rond and Miller, 2005)]. Some of this competition is an impediment to the collaborative nature of research, and the community as a whole could work much more efficiently and reduce research cost if free and open principles were extended as much as possible (respecting ethical and legal constraints). In order to advance more *efficiently* our understanding of the brain, we need to shift our scientific culture away from silos of domain expertise to a more collaborative, distributed network of information exchange; a shift from the wet lab to the web lab.

## Author Contributions

All authors listed have made a substantial, direct and intellectual contribution to the work, and approved it for publication.

### Conflict of Interest Statement

The authors declare that the research was conducted in the absence of any commercial or financial relationships that could be construed as a potential conflict of interest.

## Footnotes

^1^http://afqvault.org

^2^https://www.braininitiative.nih.gov/funded-awards/openneuro-open-archive-analysis-and-sharing-brain-initiative-data

^3^https://www.incf.org/node/295

^4^https://wellcome.ac.uk/funding/guidance/guidelines-good-research-practice

^5^https://github.com/bids-standard/bids-starter-kit

^6^http://www.reproducibleimaging.org/module-FAIR-data/00-Introduction-to-Module/

^7^https://www.github.com

^8^https://github.com/cmadan/openmorph

^9^https://www.github.com

^10^https://docker.com

^11^https://brainlife.io

^12^https://www.braincode.ca/

^13^www.plot.ly

^14^https://mybinder.org/

^15^https://notebooks.azure.com

^16^https://colab.research.google.com/notebook

^17^https://drive.google.com

^18^https://support.office.com/en-us/article/collaborate-on-word-documents-with-real-time-co-authoring-7dd3040c-3f30-4fdd-bab0-8586492a1f1d

^19^https://paperpile.com/

^20^https://www.overleaf.com

^21^https://www.authorea.com

^22^https://www.github.com

^23^https://travis-ci.org

^24^https://www.circleci.com

^25^https://gitlab.com

^26^https://www.github.com/nipy/nipype

^27^https://www.github.com/nipy/dipy

^28^https://www.github.com/nilearn/nilearn

^29^https://open-brain-consent.readthedocs.io/en/stable/

^30^https://archive.org/

^31^https://zoom.us/

^32^https://www.webex.com/

^33^https://www.bluejeans.com/

^34^https://www.skype.com/en/

^35^https://hangouts.google.com/

^36^https://appear.in

^37^https://www.gotomeeting.com/

^38^https://trello.com

^39^https://asana.com/

^40^https://www.researchtrends.com/issue-31-november-2012/force11-gains-momentum-creating-the-future-of-research-communications-and-e-scholarship/

^41^https://beallslist.weebly.com/standalone-journals.html

^42^https://d3js.org/

^43^https://github.com/rii-mango/papaya

^44^https://github.com/xtk/X

^45^http://roygbiv.mindboggle.info

^46^https://yeatmanlab.github.io/AFQBrowser-demo

^47^https://www.openanatomy.org/

^48^https://stenci.la/

^49^https://web.hypothes.is/

^50^https://www.labroots.com/virtual-event/neuroscience-2018

^51^https://medium.com/@cMadan/on-the-benefits-of-twitter-5af59158e4e2

^52^http://www.brainhack.org/global2018/

^53^https://www.miccai.org/

^54^https://www.aldacenter.org/aklc

^55^www.repronim.org

^56^https://www.coursera.org/learn/functional-mri

^57^https://www.edx.org/course/fundamentals-biomedical-imaging-magnetic-epflx-fndbioimg2x-0

^58^https://www.youtube.com/channel/UCZ7gF0zm35FwrFpDND6DWeA

^59^https://www.youtube.com/channel/UCQ8y5WCi5yAgDFxLmh2MJyg/videos

^60^https://neurostars.org

^61^https://stackoverflow.com

^62^https://arxiv.org/abs/1611.04135

^63^https://webfoundation.org/about/vision/history-of-the-web/
